# Stigma in development and evaluation of web-based weight loss programs? Characteristics of study samples - a systematic review

**DOI:** 10.1192/j.eurpsy.2025.1433

**Published:** 2025-08-26

**Authors:** N. Khorikian-Ghazari, A. Hasan, J. Grimmer, I. Papazova

**Affiliations:** 1 Department of Psychiatry, Psychotherapy and Psychosomatics, University of Augsburg, Medical Faculty; 2 DZPG (German Center for Mental Health), partner site München/Augsburg, Augsburg, Germany

## Abstract

**Introduction:**

People with a severe mental illness (SMI) have a higher prevalence of obesity than the general population. Weight gain is one of the main adverse side effects of antipsychotic treatment, leading to health complications, higher mortality and low treatment adherence in people with SMI. E-Health programs are increasingly used for weight loss.

**Objectives:**

This systematic review aims to analyze target groups and the inclusion and exclusion criteria in evaluation studies of such programs, with a focus on the exclusion of individuals with SMI and the resulting gap in healthcare.

**Methods:**

The systematic search consisted of studies in English using the databases PUBMED, PsychInfo and Cochrane Database, published between January 1, 2003 - February 29, 2024. Exclusion criteria were studies with a focus on programs other than weight loss or combined interventions. Further, we used the search terms: web-based, app* OR artificial intelligence* OR software OR online OR machine learning* OR digital* OR Internet AND weight loss OR body mass index OR weight reduction* OR body weight OR waist circumference OR obesity* OR BMI OR weight. The database search identified 4560 records, of those 633 duplicates were removed. The remaining 3927 records were further screened and 3584 were excluded, 343 full-text articles were assessed for eligibility. 117 full text articles were excluded with reasons and finally 226 studies were included in qualitative synthesis.

**Results:**

Preliminary results showed that app recommendations were reported in only 44 out of 226 studies. Additionally, analysis of target groups for recommended apps indicated that most were designed for individuals with diabetes, closely followed by apps targeting maternity, hypertension or cardiovascular diseases and corporate health management. Notably, none of the apps were specifically designed for people with a mental illness. Moreover, a comparison of the frequency of exclusion criteria for mental vs. somatic vs. chronic illnesses in general resulted in: 77:54:105. The terminology used for exclusion specifically for people with a mental illness was often general (e.g. “mental impairments”, “any psychological problem”, “unstable emotional condition”).

**Conclusions:**

The results point to deficits in the area of app recommendation and in sample selection. Specifically, people with a mental illness were most frequently excluded using vague terminology. Furthermore, there is an enormous lack of such programs for people with SMI. Knowledge about such gaps is crucial in order to avoid inadequate care for vulnerable groups. Excluding people with SMI from the development and subsequent evaluation of such programs may not adequately address their challenges and may lead to additional frustration experienced by this group due to inadequate app recommendations. The results suggest a potential stigmatization.

**Disclosure of Interest:**

N. Khorikian-Ghazari: None Declared, A. Hasan Consultant of: Rovi, Recordati, Otsuka, Lundbeck, AbbVie, Teva and Janssen-Cilag, Speakers bureau of: Janssen-Cilag, Otsuka, Recordati, Rovi, Boerhinger-Ingelheim and Lundbeck, J. Grimmer: None Declared, I. Papazova: None Declared

